# Interval Hysterectomy for Placenta Percreta – a Case Report

**DOI:** 10.5195/cajgh.2019.345

**Published:** 2019-02-05

**Authors:** Mohammad Sazzadul Huque, Mini Ravi

**Affiliations:** 1 College of Medicine, Gulf Medical University, United Arab Emirates; 2 Mafraq Hospital, United Arab Emirates

**Keywords:** Placenta Percreta, Interval Hysterectomy, Case Report, Complications of Placenta Percreta

## Abstract

**Introduction:**

Placenta percreta is an abnormality of placentation where it invades the serosa and can go beyond it. Complications include massive hemorrhage, bladder dysfunction, and severe infections during delivery. The aim of this study is to report a complex case of placenta percreta managed by interval hysterectomy.

**Case presentation:**

Pre-operative: 34 years old patient with previous three cesarean sections was followed in antenatal clinic. She came with repeated bouts of vaginal bleeding at 30–31 weeks. At 32 weeks and 4 days classical cesarean section was done with placenta left in situ. Prophylactic bilateral internal iliac artery balloon was inserted. Post cesarean section, uterine artery embolization was performed. Post-operative: Clinical features of pulmonary embolism (PE) developed about 4 hours later. Post-Operative Day 13: Total abdominal hysterectomy was done. After few days of discharge, the patient presented to the emergency department with shortness of breath. She was consequently diagnosed with chronic pulmonary embolism and treated with warfarin.

**Conclusion:**

This is a case of placenta percreta managed by interval hysterectomy. However, the most widely accepted method of management is cesarean hysterectomy. In this case, interval hysterectomy was done due to the possibility of bladder invasion by placenta, to decrease the amount of blood loss and to reduce the number of days stayed in hospital. Appropriate management for the patient must be personalized, whether it is by cesarean hysterectomy or interval hysterectomy, as each has risks and benefits.

## Introduction

Placenta accreta comes under a broad category of abnormal adherent placenta and is classified into three different entities: (1) Placenta accreta vera, where placenta invades the decidual layer of the myometrium. (2) In placenta increta, placental villi invade more deeply within the myometrium.^1^ (3) Placenta percreta is diagnosed when the placenta invades up to the serosa and can go beyond it.^2^ Placenta accreta is diagnosed in about 1:533 pregnancies where 75%–80% are placenta accreta vera, 17% placenta increta, and remaining 5% are placenta percreta. Overall, the incidence of placenta percreta is extremely low but the appearance of this rare disorder is increasing due to increase number of cesarean deliveries being performed in the past few years globally.^3^,^4^

Placenta percreta is considered one of the most severe forms of placenta accreta. It is a potentially life-threatening condition with the risk of severe maternal morbidity and mortality. In cases where placenta precreta is complicated by bladder invasion, mortality rates can be as high as 9.5% in mothers and 24% in newborn.^5^ Both diagnostic methods, sonography and MRI, have good sensitivity and specificity for prenatal diagnosis of placenta accreta.^4^ According to American College of Obstetricians and Gynecologists, widely acknowledged method to manage placenta accreta spectrum is by performing cesarean hysterectomy, in which the placenta is left in situ after delivery of the fetus because any attempts to remove the placenta is associated with substantial danger of hemorrhage.^6^ Another method of management that can be considered for placenta percreta is interval hysterectomy. In general, patients who have placenta accreta are more likely to have a caesarean section (AOR: 4.6). They are at an increased risk for being admitted to intensive care unit (ICU)/high dependency unit (AOR: 46.1) and to have a hysterectomy (AOR: 209.0). Births are expected to be preterm with a high level neonatal ICU admission and resuscitation needs.^7^ The median expected blood loss at time of cesarean hysterectomy for patients with placenta accreta has been reported to be 3 liters and the mean transfusion requirement of 5 units of packed red blood cells.^8^ In addition to higher estimated blood loss, placenta percreta patients are also at an increased risk of bladder and ureteral injury.^9^ Conservative management where the uterus and the placenta are left in-situ at time of cesarean delivery was shown to be associated with a reduction in blood loss in patients with placenta percreta, decreased need for transfusion, and less incidence of disseminated intravascular coagulation through uterus involution.^8^,^10^

We are reporting this rare case to increase the awareness about placental percreta and to share our experiences with the interval hysterectomy as a treatment modality. This is a very interesting case because it shows the complete picture of the case from antepartum history to operative details and discharge information. It also includes information of treatment that took place after discharge. It highlights the list of complication one can anticipate in a case of placenta percreta.

## Case presentation

### Antepartum history

**Figure 1:**
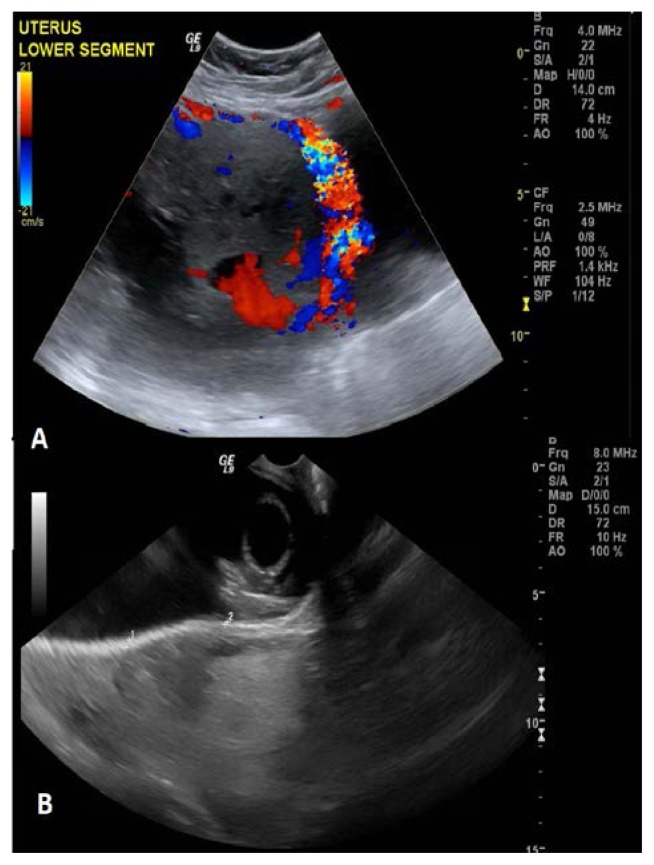
Clinical images: A. Doppler Ultrasound and B. Ultrasound of the Uterine Lower Segment

34 years old patient, Gravida 6 Parity 3, previous 2 miscarriages (18 weeks & 12 weeks), was seen first at 23 weeks 4 days of pregnancy. She had undergone previous 3 cesarean sections and an evacuation of retained products of conception by curettage in 2013 for partial hydatidiform mole. At 27 weeks 5 days, she was admitted for vaginal bleeding. On further evaluation by ultrasound (Figure 1), the diagnosis of placenta percreta was made (later confirmed by MRI). At 29 weeks, she had constipation with 2 episodes of urinary retention and she was put on continuous bladder drainage. She developed urinary tract infection and treated with appropriate antibiotics based on culture sensitivity. She continued to have repeated bouts of vaginal bleeding of varying amounts and severe constipation from 31 weeks of gestation.

### Operative history

At 32 weeks 4 days, patient underwent cystoscopy, which had shown signs of cystitis with no definite infiltration. She underwent classical cesarean section under combined anesthesia (Epidural + General). The umbilical cord was tied near insertion and the placenta was left in situ because there was no spontaneous separation. Then, the uterus was closed. Prophylactic temporary bilateral internal iliac artery balloons were inserted and inflated earlier. Uterine artery embolization was performed post cesarean section and selective angiograms confirmed adequate positioning. The patient required large volume of particles and still had incomplete embolization with the lower part of the uterus still showing some unblocked branches on both sides.

### Post-operative course

Post-operatively, she was transferred to labor ward and within 4 hours, she developed clinical features of pulmonary embolism (PE). Some of her symptoms included drop in O_2_ saturation to 81%, tachycardia, chest pain, peripheral cyanosis, and signs of respiratory distress. Then, she was transferred to ICU and was initiated on heparin infusion. On chest X-ray, she had no atelectasis, pneumothorax, or pleural effusion. An immediate CT scan did not show any PE. There was no Doppler evidence of venous thrombosis in the femoral and popliteal venous systems. Later on day 1 post-operative, she had focal patchy consolidation left base and was started on parenteral meropenem, linezolid and fluconazole for the next 5 days. She had two consecutive CT scans on post-operative on days 2 and 3, which were negative. On ECG, there was right heart strain. She was now on enoxaparin. On the post-operative day 5, she was prescribed parenteral piperacillin-tazobactam for 5 days and she was shifted out of ICU next day. She had 500ml vaginal bleeding on the 9^th^ post-operative day. 2 units PRBC were transfused. She was switched to oral cefuroxime and metronidazole and planned to continue on long-term low dose antibiotic. On post-operative day 11, she received methotrexate. On day 12, the MRA had shown the placenta was still enhancing with some areas of infarct and separation, fluid collection in the uterine cavity (present from day 1 post op, not increasing), with large ovarian veins, hugely distended and extensive pelvic varices, R>L, extensive collaterals. Her CRP was 12.7 mg/L.

### Operative details

On post-operative day 13, she underwent total abdominal hysterectomy. Intraoperatively, the bladder was densely adherent, drawn up, with large vessels in the broad ligament. The lower segment was bulging due to the presence of the placenta. The uterus was about 24 weeks’ size with adherent omentum. There was 100 mL of old blood in the cavity and the placenta was partially infarcted. The total blood loss was 2000 mL.

### Post-hysterectomy period

Post-operatively, she was in ICU for 2 days receiving anticoagulation treatment (bridging treatment with enoxaparin + warfarin) and patient controlled analgesia. She had a bout of severe cough on day 4 and loose motions on day 5. She was diagnosed with vault hematoma, which was retro-vesical, about 120 ml in volume, treated conservatively. On day 10 she had been discharged from the hospital.

She presented to the ER on the post-operative day 16 and was diagnosed with chronic pulmonary embolism. Patient had a pulmonary embolus within the right middle lobe pulmonary artery; areas of sub-segmental embolus within the right lower lobe pulmonary arteries. She had no pleural effusions or consolidation and no mediastinal lymphadenopathy. She was readmitted for 4 days. She was started on therapeutic enoxaparin + warfarin. She was continued on 6 mg warfarin for 4 weeks after discharge.

## Discussion

In the field of abnormal placentation, placenta percreta is the most uncommon and most dangerous among them. The diagnosis of this condition can be achieved during pregnancy by ultrasound and/or magnetic resonance imaging. The main aim of the therapy should be to minimize the blood loss by doing a hysterectomy, or by preventing the elimination of the placenta at the time of delivery, or via methotrexate regiment for the ablation of the remaining placenta in the post-delivery period.^11^ In USA, the most common method of threatment for placenta accreta is cesarean hysterectomy. The complications of cesarean hysterectomy are bladder and/or ureters injury, severe hemorrhage and maternal demise. Placental retention with uterine conservation during the time of delivery and interval hysterectomy are the other options available for stable patient. These techniques can result in decreased amount of blood loss and bladder/other organs resection.^12^

Previous studies report cases of invasive placentation that were managed by interval hysterectomy. One case had placental invasion up to the anterior abdominal wall. By delaying patient’s hysterectomy and using uterine artery embolization, patient was able to undergo the hysterectomy and bladder resection with less morbidity when compared to the scenario when procedure would have been performed during cesarean delivery, which was already made difficult by the immense hemorrhage.^12^ In the case series by Wong et al, eight suspected cases of abnormal placentation were managed. In three cases, the placentas were separated from the uterus with minimal difficulty. In two cases of placenta percreta without invasion of bladder, cesarean hysterectomy was done. In the remaining three cases of percreta with bladder invasion, the entire placenta was left in-situ. Resolution occurred in two of them, over a period of 8 and 12 months respectively. The last one had post-operative course complication with deep vein thrombosis and disseminated intravascular coagulation. This patient underwent hysterectomy with preoperative uterine artery embolization, inferior vena cava filter placement and ureteric stenting. Hence, the conservative management is potentially safe and attractive alternative to the other modalities. However, cautious and vigilant patient selection with individualized assessment is needed.^13^

In our case, we chose interval hysterectomy because of possible bladder invasion by placenta, to decrease the amount of blood loss, and to reduce the hospital stay. In another study of 93 patients with abnormal placentation, 20 patients have been diagnostically confirmed to have placenta percreta. Out of these women, 11 underwent immediate hysterectomy; 9 underwent interval hysterectomy. Median approximate blood loss for women who underwent immediate hysterectomy (2.8L) was significantly higher compared to interval hysterectomy (1L). Median duration of stay for the immediate hysterectomy was 15 days compared to 7 days for the interval hysterectomy. However, there was a trend towards increased rate of infection with interval hysterectomy.^14^

In conclusion, this case highlights the need to investigate various management options for patients with placenta percreta. While it is a rare diagnosis, it is increasing in incidence. It should be highlighted that cesarean section should be reserved to the patients for whom it is indicated. In addition, it is difficult, risky, and expensive to manage such cases. Nevertheless, proper management for the patients must be individualized whether it is cesarean hysterectomy or interval hysterectomy, as each option has its own risk and benefits, and must be performed with caution.
